# The Book World of Medicine and Science

**Published:** 1894-02-10

**Authors:** 


### The Book World of Medicine and Science.

### BOOK REVIEWS.

Asiatic Cholera, its History, Pathology, and Modern Treatment. By A. J. Wall, M.D., F.R.C S. (London: H. K. Lewis).

Asiatic cholera is always of interest to the philosophic medical historian, but at the present moment it is also a subect of immediate practical importance both to the medical profession and to the public. Dr. A. J. Wall, who has spent many years in India, and who is now enjoying the leisure of the retired army surgeon, has done well to improve that leisure in placing before the profession the intelligent and mature convictions of many years of experience and careful study in connection with Asiatic cholera. The history of a disease like cholera is a guide to its causation, nature, and treatment. Dr. Wall presents us with the history of the subject in a very condensed but thoroughly comprehensive form. He does not believe, as do Mr. Ernest Hart and some others, that cholera is exclusively a water-borne disease, and that consequently the maintenance of an abundant supply of pure drinking water is all that is required to prevent alarming epidemics. Now this is clearly a point of fundamental importance, and one would like exceedingly to have it cleared up one way or another. That cholera is a disease which is carried by impure water no experienced scientist for a moment doubts; but that it can never be spread in epidemic proportions by any other means than that of water contamination is a proposition which is certainly not proved. Of course we all know that the excreta of cholera patients are virulent sources of infection, as indeed they must be if the bacillary origin of the disease is admitted. But that even the excreta, coupled with the water contamination theory, exhaust the possibilities of epidemical contagion we are not prepared to admit, neither is Dr. A. J. Wall.

On the question of the etiology of cholera, as well as on that of its general propagation, Dr. Wall has ventured to think for himself, and he has assailed the too readily accepted dogmas of Koch with much force of reasoning and with many striking facts. The book is scientific and practical, and is well worthy of the study both of the pure scientist and of the practical clinician. There is a very foolish tendency on the part of laboratory medical men to run after one another, and to swear by names. But a little actual fact and bedside experience are worth a great deal of mere laboratory theorising. Dr. Wall's book is an excellent set-off against the ill-digested and crude speculations of those youthful researchers who speak what they do not know, and testify what they have not seen.

### FEBRUARY REVIEWS.

Sir William des Vœux, late Governor of Hong Kong, contributes a vigorous defence of opinion to the Nineteenth Century in "A Letter to Opium Commission." He argues that the population of Hong Kong smokes more opium than any other of equal numbers in the world, and yet remains active, industrious, and healthy in spite of the most disadvantageous sanitary conditions. He is even of opinion that the large quantities of opium consumed by the Chinese stimulates their activity, and preserves them from infection and from the effects of overcrowding, while he considers there is grave reason to fear the introduction of alcohol leading to far graver evils should the opium trade be suppressed. Sir Douglas Galton pleads the cause of the "Feeble-minded Children," whose right education is now exercising the wits of the British Medical Association, the Royal Statistical Society, and the Charity Organisation. Some 10,000 children in the London school area alone have been found to present signs of imperfect development, necessitating exceptional treatment, and it is greatly to be desired that a definite effort should be made to furnish them with the special training without which they must drift into idleness or crime.

The Fortnightly has some very interesting recollections of Professor Tyndall, contributed by Mr. Herbert Spencer. The Professor's unappeasable appetite for debate proved on more than one occasion too much for Mr. Spencer's physical powers. "'Do you believe in matter'? was a question which he propounded just as we were about to bid one another good night after a day's continuous talking . . . persistence in this kind of thing was out of the question, and I had to abridge my stay. Once more the like happened when, after the meeting of the British Association at Liverpool, we adjourned to the Lakes. There was almost unceasing discussion as we rambled along the shore of Windermere, or walked up to Rydal Mount. Tyndall's intellectual vivacity gave me no rest; and after two utterly sleepless nights I had to fly." The ceaseless "intellectual activity" disturbed not only the rest of his guests, but inflicted on himself that insomnia by which his after life was troubled, and by which his powers of work seem to have been materially diminished. Mr. Henry Forbes, in a very able paper, "Antarctica: A Vanished Austral Land," shows cause for the belief that an extensive continent existed in the Southern Hemisphere, on which many forms of terrestrial life originated, and had there the original centre of their development and dispersal.

Most of the Reviews have some notice of the recent Life of Dean Stanley which Mr. Prothero and Dean Bradley have now brought to a conclusion, and in nearly every case a feeling of disappointment is apparent. Sir Mountstuart E. Grant Duff, in his notice of the biography for the National Review, thinks its disappointing characteristics are mainly due to the controversial element. Stanley passed much of his life in fighting with wild beasts at Ephesus. We who lived through those times know all about those beasts, and have no wish to read once more the record of their names. The writer's suggestion that in future editions the history of Stanley's controversies should be a good deal abridged is likely to find favour with most readers.

In the Contemporary the Philosophy of Crime is ably dealt with by Mr. W. S. Lilly. Mr. Pater's article on the Age of Athletic Prizemen will bear a good deal of reading, representing as it does a wonderful store of learning in small compass. "An Old Fogey" has some pertinent remarks to make on "The Young Men," but if the evidence of style can be accepted the "old fogey" can be identified as one who a very short time back was counted himself among the band whom he here gracefully sets to rights.

### Books Received.

H. K. Lewis.

